# PAR Genes: Molecular Probes to Pathological Assessment in Breast Cancer Progression

**DOI:** 10.4061/2011/178265

**Published:** 2011-01-24

**Authors:** Beatrice Uziely, Hagit Turm, Myriam Maoz, Irit Cohen, Bella Maly, Rachel Bar-Shavit

**Affiliations:** ^1^Departments of Oncology, Hadassah-University Hospital P.O. Box 12000, Jerusalem 91120, Israel; ^2^Departments of Pathology, Hadassah-University Hospital P.O. Box 12000, Jerusalem 91120, Israel

## Abstract

Taking the issue of tumor categorization a step forward and establish molecular imprints to accompany histopathological assessment is a challenging task. This is important since often patients with similar clinical and pathological tumors may respond differently to a given treatment. Protease-activated receptor-_1_ (PAR_1_), a G protein-coupled receptor (GPCR), is the first member of the mammalian PAR family consisting of four genes. PAR_1_ and PAR_2_ play a central role in breast cancer. The release of N-terminal peptides during activation and the exposure of a cryptic internal ligand in PARs, endow these receptors with the opportunity to serve as a “*mirror-image*” index reflecting the level of cell surface PAR_1&2_-in body fluids. It is possible to use the levels of PAR-released peptide in patients and accordingly determine the choice of treatment. We have both identified PAR_1_ C-tail as a scaffold site for the immobilization of signaling partners, and the critical minimal binding site. This binding region may be used for future therapeutic modalities in breast cancer, since abrogation of the binding inhibits PAR_1_ induced breast cancer. Altogether, both PAR_1_ and PAR_2_ may serve as molecular probes for breast cancer diagnosis and valuable targets for therapy.

## 1. Introduction

The classification of a tumor differentiation level is routinely based on histopathological criteria whereby poorly differentiated tumors generally exhibit the worst prognoses. However, the underlying molecular pathways that regulate the level of breast tumor development are as yet poorly described. Until now the pathological tissue criteria that entail tissue traits have not been defined by an appropriate set of genes. A challenging task is to take the issue of breast tumor categorization a step forward and establish molecular imprints to accompany histopathological assessment. This is important since often patients with similar clinical and pathological tumors may have a markedly different outcome in response to a given treatment. These differences are encoded by and stem from the tumor genetic profile [1]. Individual gene signature may complement or replace the traditional pathological assessment in evaluating tumor behavior and risk. This is the basis for optimizing our approach to personalized care whereby genomic finger prints may refine the prediction of the course of disease and the response to treatment [2]. Oncotype Dx is a clinically validated and widely used multigene assay (there are also other commercially available gene panels such as Mammaprint; Agendia Amsterdam, Netherland, and THEROS H/I; Biotheranostics, San Diego, CA), that quantifies the likelihood of breast cancer recurrence. This gene profile has been developed specifically for women with hormone receptor-positive (estrogen and progesterone receptor; ER, PR) and lymph node-negative disease. The gene profile consists of 21 genes that are associated with disease recurrence. Sixteen are cancer-related genes and 5 serve as reference genes. This gene panel is used to calculate the recurrence score (RS), a number that correlates with the specific likelihood of breast cancer recurrence within 10 years from the original diagnosis. Therefore, an ongoing goal is to identify important genes that play a central part in breast cancer biology and determine their relative function during the course of breast cancer progression [3]. Identification of these genes will significantly contribute to the prospect of treatment making choices.

Protease-activated receptor-_1_ (PAR_1_), a G protein-coupled receptor (GPCR), is the first and prototype member of the mammalian PAR family consisting of four genes. The activation of PAR_1_ involves the release of an N-terminal peptide and the exposure of an otherwise hindered ligand, resulting in an exclusive mode of activation. This mode of activation serves as a general paradigm for the entire PAR family [4–6]. While a well-known classical observation points to a close link between hyperactivation of the coagulation system and cancer malignancies, the molecular mechanism that governs procoagulant tumor progression remains poorly defined [7–10]. Thrombin is a main effector of the coagulation cascade. In addition to cleaving fibrinogen, it also activates cells through at least three PARs: PAR_1_, PAR_3_, and PAR_4_. In contrast, PAR_2_ is activated by multiple trypsin-like serine proteases including the upstream coagulant proteases VIIa—tissue factor (TF) and Xa, but not by thrombin. It is now becoming well established that human *Par_1_, hPar*_1, _ plays a central role in epithelial malignancies [13, 14, 16]. PAR_2_, the second member of the family, is also emerging with central assignments in breast cancer [11, 12]. High levels of *hPar*_1_ expression are directly correlated with epithelia tumor progression in both clinically obtained biopsy specimens and a wide spectrum of differentially metastatic cell lines [13, 14]. PAR_1_ also plays a role in the physiological invasion process of placental cytotrophoblasts during implantation into the uterus deciduas [15]. Trophoblast invasion shares many features with the tumor cell invasion process. It differs, however, by the time-limited *hPar*_1_ expression, which is confined to the trophoblast-invasive period and is shut off immediately thereafter, when there is no need to invade [13]. This strongly supports the notion that the *hPar*_1_ gene is part of an invasive gene program. Surprisingly, the zinc-dependent matrix-metalloprotease 1 (MMP-1), a collagenase that efficiently cleaves extra cellular matrix (ECM) and basement membrane components, has been shown to specifically activate PAR_1_ [16]. PAR_1_-MMP1 axis may thus provide a direct mechanistic link between PAR_1_ and tumor metastasis. The mechanism that leads to *hPar*_1_ gene overexpression in tumor is yet unclear and under current extensive investigation. Although the impaired internalization of PAR_1_ that results with persistent signaling and invasion was previously suggested for several breast cancer lines [17], an imbalanced expression between *hPar*_1_ repressors and activators was proposed, suggesting transcriptional regulation [18]. We found that the mechanism of *hPar*_1_ overexpression involves enhanced transcriptional activity, whereby enhanced RNA chain elongation takes place in the aggressive cancer cells as compared with the nonaggressive, low metastatic potential cells [19]. Indeed, we have identified the *Egr-1* transcription factor as a critical DNA-binding protein eliciting *hPar*_1_ expression in prostate cancer cells and the *wt* p53 tumor suppressor as an *hPar*_1_ transcription repressor [19, 20]. The *wt* form of p53 thus acts as a fine-tuning regulator of *hPar*_1_ in cancer progression.

## 2. Prognostic Parameters of PARs

The PARs act as delicate sensors of extra cellular protease gradient to allow the cells to respond to a proteolytically modified environment. The fact that PAR_1_ gene and protein overexpression are associated with the aggressiveness of a tumor, *in vivo*, reflect its potential role in cancer dissemination. Furthermore, it assigns PAR_1_ as an attractive target for anticancer therapy. On the other hand, the release of an N-terminal peptide during activation and the exposure of an otherwise cryptic internal ligand in PARs endow these receptors with the opportunity to serve as a “*mirror-image”* index reflecting in body fluids the level of PARs on the surface of cancer cells. Hence, PAR_1_ and PAR_2_ peptides in the blood directly imitate PAR expression serving as a faithful indicator for the extent of cancer progression. While the overexpression of both PAR_1_ and PAR_2 _ takes place on the surface of cancer cells that are being constantly turned over in the body, yet there is no current information as to the half -life of the released peptides. It is envisioned that measuring the level of released peptides may underline the severity of cancer. Another aspect is that the followup levels of PAR_1_-released peptides may be instrumental in demonstrating the effectiveness of a given treatment. For example, determining the level of the released PAR_1_ and PAR_2_, through repeated measurements in the blood stream, may serve as a base line for a patient, and a sensitive indicator for response to a treatment. If the released PAR peptides are becoming gradually low and finally disappear, it may reassure that the tumor is indeed regressing until finally the cancer disappears. In contrast, if the level remains unchanged, it may indicate that the tumor is progressing despite of a given treatment. A critical aspect, however, that needs to be addressed is the prospect of high released PAR_1&2_ peptides present during inflammation [21, 22]. Therefore, the repeated *followup* of PAR released peptides is necessary for the purpose of demonstrating that during inflammation the high PAR-released peptide level is transient and disappears when the inflammatory response is over. In contrast, in the case of a tumor, the level of PAR-released peptides remains constantly high. The relative contribution of PAR_1_ versus PAR_2_ during the process of tumor progression is as yet unknown and is under current investigation. One approach to decisively address this issue is by immunohistological staining (of anti-PAR_1_ and anti-PAR_2_ antibodies, separately) utilizing tissue microarray biopsy specimens on a large pool of primary breast cancer biopsy specimens representing invasive carcinoma. Such analysis will determine the relative percentage of PAR-positive individuals in a given cancer patient pool. Whether PARs join the triple negative population (ER-, PR-, and Her-2/Neu, an indicator of disease aggressiveness)—or perhaps stands independently as a prognostic marker—needs to be evaluated.

## 3. PARs as Target for Therapy

Importantly, PAR_1_ cellular trafficking and signal termination appear to occur in a different mode than other GPCRs. Instead of recycling back to the cell surface after ligand stimulation, activated PAR_1_ is sorted to the lysosomes where it is degraded [23, 24]. While cellular trafficking of PAR_1_ impinges on the extent and mode of signaling, the identification of individual PAR_1_ signaling partners and their contribution to breast cancer progression remain to be elucidated.

 We have adopted the approach of utilizing a truncated form of *hPar*_1_ gene devoid of the entire cytoplasmic tail to demonstrate the significant role of PAR_1_ signaling in breast tumor progression. This was demonstrated in a xenograft mice model of mammary gland tumor development*, in vivo* [25]. Along this line of evidence, we have identified PAR_1_ C-tail as a scaffold site for the immobilization of signaling partners. In addition to identifying key partners, we have determined the hierarchy of binding and established a region in PAR_1_ C-tail critical for breast cancer signaling. This minimal binding domain may provide a potent platform for future therapeutic vehicles in treating breast cancer. The above-described outcome is a brief summary of the detailed experimental approach illustrated bellow. 

 The functional outcome of MCF7 cells overexpressing various *hPar*_1_ constructs *in vivo* was assessed by orthotopic mammary fat pad tumor development. MCF7 cells overexpressing either persistent *hPar_1_ Y397Z* or *wt hPar*_1_ constructs (e.g., MCF7/*Y397Z hPar*_1_; MCF7/*wt hPar*_1_) markedly enhanced tumor growth *in vivo* following implantation into the mammary glands, whereas MCF7 cells overexpressing truncated *hPar*_1_, devoid of the entire cytoplasmic tail, behaved similarly to control MCF7 cells in vector-injected mice, which developed only very small tumors. The tumors obtained with MCF7/*wt hPar*_1_ and MCF7/*Y397Z hPar*_1_ were 5 and 5.8 times larger, respectively, than tumors produced by the MCF7/empty vector-transfected cells. Histological examination (H&E staining) showed that while both MCF7/*wt hPar*_1_ and MCF7/*Y397Z hPar*_1_ tumors infiltrated into the fat pad tissues of the breast, the MCF7/*Y397Z hPar*_1_ tumors further infiltrated the abdominal muscle. In contrast, tumors produced by empty vector or truncated *hPar*_1_-transfected cells were capsulated, with no obvious cell invasion. Tumor growth can also be attributed to blood vessel formation [26, 27]. The *hPar*_1_-induced breast tumor vascularization was assessed by immunostaining with antilectin and anti-CD31 antibodies, showing that both MCF7/*Y397Z hPar hPar*_1_ and MCF7/*wt hPar*_1_ tumors were intensely stained. In contrast, only few blood vessels were found in the small tumors of empty vector or truncated *hPar*_1_. Thus, both MCF7/*wt hPar_1 _* and MCF7/*Y397Z hPar*_1_ cells were shown to effectively induce breast tumor growth, proliferation, and angiogenesis, while the MCF7/truncated *hPar*_1_ and MCF7/empty vector-expressing cells had no significant effect. This experimental results highlight the significance of PAR_1_ signaling in PAR_1_-induced breast cancer progression.

## 4. Antibody Array for Protein-Protein Interactions Reveals Signaling Candidates

Next, in order to identify specific PAR_1_ signaling components, the following approach was utilized. To detect the putative mediator(s) linking PAR_1_ to potential signaling pathway, we examined a custom-made antibody-array membranes. When aggressive breast carcinoma MDA-MB-435 cells (with high *hPar*_1_ levels) were incubated with the antibody-array membranes before and after PAR_1_ activation (15 minutes), the following results were obtained. Several activation-dependent proteins which interact with PAR_1_, including ICAM, c-Yes, Shc, and Etk/Bmx, were identified. Of these proteins, we chose to focus here on Etk/Bmx and Shc. 

The epithelial tyrosine kinase (Etk), also known as Bmx, is a nonreceptor tyrosine kinase that is unique by virtue of being able to interact with both tyrosine kinase receptors and GPCRs [28]. This type of interaction is mainly attributed to the pleckstrin homology (PH) which is followed by the Src homology SH3 and SH2 domains and a tyrosine kinase site [29]. Etk/Bmx-PAR_1_ interactions were characterized by binding of lysates exhibiting various *hPar_1 _* forms to GST-PH-Etk/Bmx. While *Y397Z hPar*_1_and *wt hPar*_1_ showed specific association with Etk/Bmx, lysates of truncated *hPar_1 _* or JAR cells (lacking PAR_1_) exhibited no binding. A tight association between the PAR_1_ C-tail and Etk/Bmx was obtained, independent of whether *wt* or kinase-inactive Etk/Bmx (KQ) was used [29, 30]. 

## 5. Hierarchy of Binding

Next, we wished to determine the chain of events mediating the signaling of PAR_1_ and the binding of Shc and Etk/Bmx to PAR_1_ C-tail. Shc is a well-recognized cell signaling adaptor known to associate with tyrosine-phosphorylated residues. To this end, analysis of MCF7 cells that express little to no *hPar*_1_ were ectopically forced to overexpress *hPar*_1_ gene. When coimmunoprecipitation with anti-PAR_1_ antibodies following PAR_1_ activation was performed, surprisingly, no Shc was detected in the PAR_1_ immunocomplex. Shc association with PAR_1_ was fully rescued only when MCF7 cells were initially cotransfected with Etk/Bmx, resulting with abundant assembly of Shc in the immunocomplex. Thus, Etk/Bmx is a critical component that binds first to activated PAR_1_ C-tail enabling the binding of Shc. Shc may bind either to phosphorylated Etk/Bmx, via its SH2 domain, or in an unknown manner to the PAR_1_ C-tail, provided that Etk/Bmx is present and is PAR_1_-bound complex. One cannot, however, exclude the possibility that Bmx binds first to Shc, and only then the complex of Etk/Bmx-Shc binds to PAR_1_.

**Figure 1:**
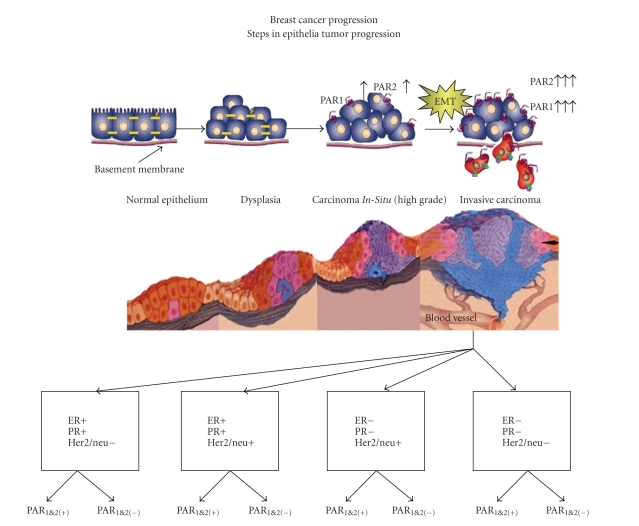
Steps in breast cancer progression. Subtypes definition of breast cancer according to ER, PR, and Her2/neu status. Additional categorization is suggested including PARs status.

**Figure 2:**
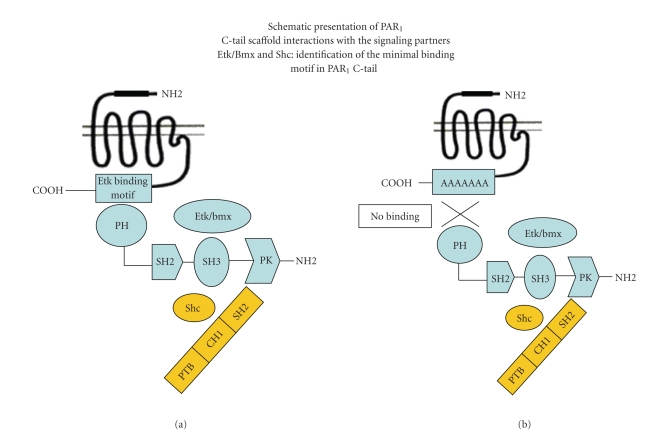
Activation of PAR_1 _ leads to the association of Etk/Bmx with PAR_1_ C-tail. This association is mediated through Etk/Bmx PH- domain enabling next the binding of Shc. The site of the “signal binding” domain (e.g., Etk/Bmx, as a prime signaling partner) in PAR_1_ has been identified. Insertion of successive replacement of A residues forming a PAR_1_ mutant incapable of binding Etk/Bmx showed impaired capabilities of PAR_1_ induced invasion and migration. This site provides therefore a platform for the development of future therapeutic medicaments in breast cancer.

 The functional consequences of the Etk/Bmx binding was further evaluated by inserting mutations to the “signal-binding” site. We prepared * hPar*_1_ constructs with successive replacement of the designated seven residues (378-384; CQRYVYS) with A, termed as *hPar*_1_-7A. This HA tagged mutant, HA-*hPar*_1_-7A, completely failed to immunoprecipitate Etk/Bmx. In contrast, in the presence of HA-*wt hPar*_1_, potent immunoprecipitation was obtained. We thus conclude that the critical region for Etk/Bmx binding to PAR_1_ C-tail resides in the vicinity of CQRYVYS. The physiological significance of PAR_1_-Etk/Bmx binding is emphasized by the following outcome. Activated MCF7 cells that express *hPar*_1_-7A mutant failed to invade Matrigel-coated membranes. In-contrast, a potent invasion was obtained by activated *wt hPar*_1_. This outcome highlights the fact that by preventing the binding of a key signaling partner to PAR_1_ C-tail, efficient inhibition of PAR_1_ pro-oncogenic functions, including the loss of epithelial cell polarity, migration, and invasion, is obtained (see Figures 1 and 2 for *wt *and mutated PAR_1_ C-tail and the ability to form a scaffold complexes with the signaling partners). Elucidation of the PAR_1_ C-tail binding domain may therefore provide a potent platform for future therapeutic vehicles in treating breast cancer. 

 The same approach may be utilized to identify a prime signaling partner for PAR_2_. This will eventually lead to characterization of a minimal PAR_2_ C-tail binding region. Generation of peptides that can enter the cells via adding Tat or penetratin, or alternatively, addition of either myristoylation, or another lipid moiety, will assist the peptides to cross the cell membrane. These peptides may prove as effective therapeutic inhibitors of PARs-induced breast cancer growth and development. Along this line of evidence, successful PAR_1_-derived peptides termed “pepducin” were developed by the group of Kuliopulos A [31]. This group has demonstrated that PAR_1_-induced breast tumor in a mouse model, *in vivo,* is blocked by the cell-penetrating lipopeptide “pepducin,” P1pal-7, which is a potent inhibitor of cell viability in breast carcinoma cells expressing PAR_1_. It has been shown that P1pal-7 is capable of promoting apoptosis in breast tumor xenografts and significantly inhibits metastasis to the lung. 

In summary, PARs may provide a timely effective challenge for developing valuable prognostic vehicles and also critical targets for therapy in breast cancer. While the PAR prognostic vehicles stem from the extracelluar portion of the receptors, we offer the intracellular C-tail site as potential targets for therapy in breast cancer. What is the relative contribution of PAR_1_ versus PAR_2_ in breast cancer tumor growth and development is yet an open question and a subject of current evaluation.

##  Conflict of Interests

The authors have declared that no conflict of interests exists.
